# Probing patterns for prognostic potential

**DOI:** 10.1038/s41398-022-01931-z

**Published:** 2022-04-21

**Authors:** Seth M. Levine

**Affiliations:** 1 Department of Psychology, LMU Munich, Leopoldstraße 13, 80802 Munich, Germany; 2 NeuroImaging Core Unit Munich (NICUM), University Hospital LMU, Nußbaumstraße 7, 80336 Munich, Germany

**Keywords:** Prognostic markers, Human behaviour, Neuroscience

Dear Editor,

In a recent article, Boehm et al. combine functional magnetic resonance imaging (fMRI) with classification-based multivariate pattern analysis [1] to investigate changes in the cognitive representations of visually presented food stimuli in anorexia nervosa (AN) patients who were acutely underweight (acAN) and in weight-recovered AN patients (recAN) [2]. The authors report that a machine learning algorithm can discriminate representations of food stimuli better than representations of neutral stimuli—in certain brain regions—within the acAN sample but not within an age-matched sample of healthy controls (HC_acAN_). Moreover, this discriminability of food-vs-neutral representations in the recAN sample does not statistically differ from that of an age-matched sample of healthy controls (HC_recAN_). The authors take these findings to suggest that cortical representations of food are altered in acAN compared to recAN, which may be indicative of altered attentional mechanisms in the presence of food in AN patients.

Such a pattern of results may also be useful as a prognostic marker, based on the authors’ regression analysis after a one-year assessment following treatment, thereby offering fMRI and cognitive neuroscience methods an additional avenue to inform the clinical domain. As such, this study is a great example of investigating cognitive processes with functional neuroimaging in a patient population and should act as a stepping stone upon which future studies can build. To this end, there are three aspects of the article that merit further discussion: caveats in the statistical inference, complementing the classification analysis with representational similarity analysis [3], and the manner in which attentional mechanisms may underlie such results.

**Fig. 1:**
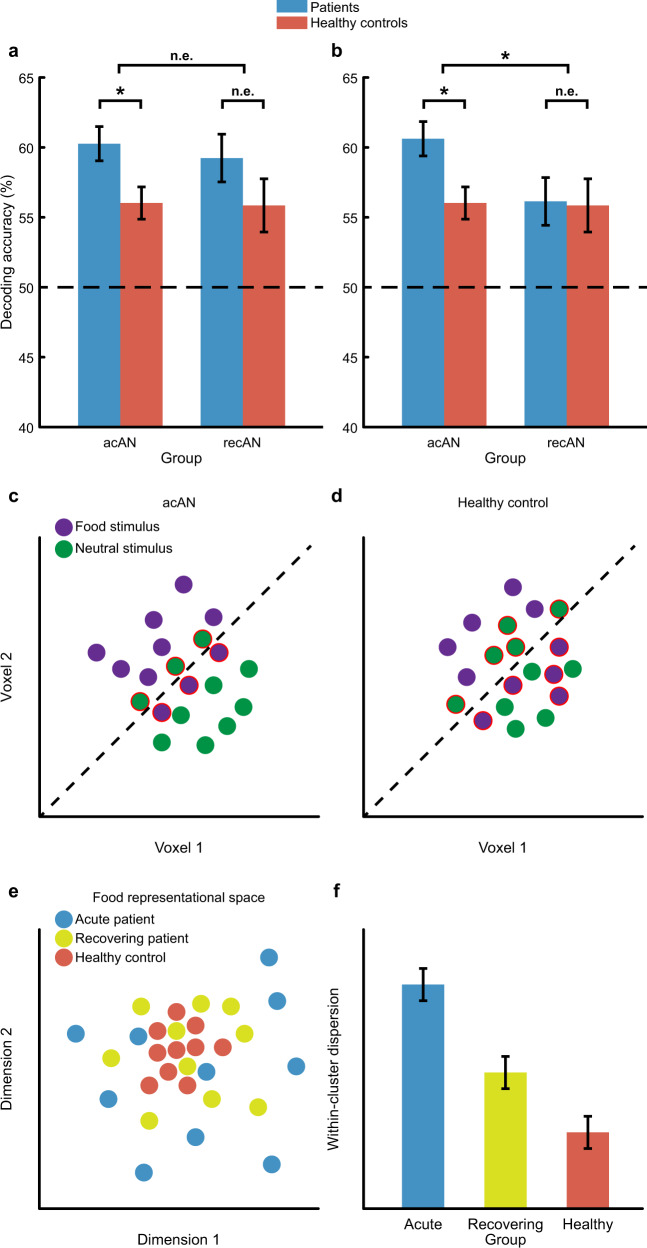
Perspectives on data interpretation. **a** Hypothetical results from a decoding analysis that would be consistent with the authors’ reported findings (i.e. evidence for a statistical difference [*] between acAN and HC_acAN_ but no effect [n.e.] discernable between recAN and HC_recAN_) but insufficient to claim a difference between these two effects. **b** Hypothetical results that would support the hypothesis put forth by the authors and provide evidence for an interaction effect, which, importantly, would be driven by a decrease in classification performance in the recAN group compared to the acAN group. **c** Simulated data that depict how activity patterns evoked by food (purple circles) and neutral (green circles) stimuli may disperse within a two-voxel space for acAN patients. The dashed line represents the hyperplane determined by a classification algorithm, which ultimately yields an accuracy of 70% (red contours depict misclassifications). **d** Same conventions as **c** but for healthy controls, in which case a classifier would fail to decode food stimuli from neutral stimuli. In both cases, the classifier indicates, at best, whether information pertaining to these two classes in such a two-voxel space is decodable but provides no additional information about the underlying distributions. **e** To complement the decoding analyses, one could directly probe the activity patterns of several groups within a given n-dimensional representational space (here visualised in two dimensions potentially following multidimensional scaling) and compare properties of their distributions. This approach would permit one to investigate potential hypotheses such as **f** whether the dispersion (i.e., the dissimilarity) of food representations increases as a function of the severity of an individual’s symptoms. All data presented in this figure were simulated.

The authors’ main finding from the multivariate analysis involves a difference in classification performance for food-vs-neutral representations when contrasting acAN patients with HC_acAN_, and that this difference in classification performance diminishes when contrasting recAN with HC_recAN_. However, the contrast that warrants the most meaningful interpretation of the results (given the scope of the study) is the interaction of these two contrasts (i.e., [acAN > HC_acAN_] > [recAN > HC_recAN_]). Reporting evidence for a difference between one set of groups (e.g., *p*_1_ < 0.05) and a lack of evidence for a difference between the other set of groups (e.g., *p*_2_ > 0.05) merely compares their effect sizes but does not directly test for the difference between the group differences [4]. Instead, demonstrating evidence for the interaction, and crucially that the interaction is driven by the contrast [acAN > recAN], would provide the most compelling evidence for the authors’ interpretation of the results (Fig. 1a, b). Additionally, given that the control samples also differed in their ages, finding that the interaction is not driven by the contrast [HC_acAN_ > HC_recAN_] would help to rule out the between-groups age-confound mentioned in the limitations section, as the authors would demonstrate that age alone is insufficient to explain potential differences between acAN and recAN. Note that this criticism does not imply that the authors’ interpretation is necessarily incorrect, but rather that the interpretation (i.e., that recAN differs from acAN) is not directly warranted from the statistical tests performed; in the best case, the patient samples differ from one another, while the control samples do not (i.e., [acAN ≠ recAN] ∩ [HC_acAN_ = HC_recAN_]), while in the worst case, the opposite pattern is observed (i.e., [acAN = recAN] ∩ [HC_acAN_ ≠ HC_recAN_]).

Regardless, exploring differences in classification performance to infer altered information processing for prognostic purposes is a shrewd application of machine learning [5]. However, given the different goals of decoding-based and encoding-based analyses [6], one can complement the classification analyses (Fig. 1c, d) with, for example, representational similarity analysis [7], thereby gleaning insight regarding how the representations are changing [8].

This strategy would be of particular interest, given the authors’ supposition that altered attentional mechanisms towards food underlie the classifier’s differential performance across groups. Previous work has shown that attention alters representational spaces to increase the categoricity of the attended feature [9]. As such, one could investigate whether neural representations of food in acAN patients are, for example, unusually dispersed (or compact) with respect to those of healthy controls or recAN (Fig. 1e, f) and, with additional encoding analyses, whether these representations tend to distribute along different dimensions underlying the food representational space. Such approaches could help to unravel how attentional mechanisms may (pathologically) affect cognitive processes related to food in acAN patients and determine whether any individual-level alterations in the representational space have additional prognostic value [10].

This correspondence aims to highlight the clever manner in which the authors combined machine learning with fMRI to investigate cognitive changes in patients with AN while simultaneously drawing attention to a few caveats/strategies in the analyses and interpretations that researchers and reviewers should take into account when designing and assessing functional neuroimaging experiments.
